# Postprandial triglyceride levels rather than fat distribution may reflect early signs of disturbed fat metabolism in Iraqi immigrants

**DOI:** 10.1186/s12944-022-01679-x

**Published:** 2022-08-04

**Authors:** Karin G. Stenkula, Lisa Esbjörnsson Klemendz, Claes Fryklund, Nils Wierup, Wathik Alsalim, Mona Landin-Olsson, Lena Trinh, Sven Månsson, Louise Bennet

**Affiliations:** 1 Department of Experimental Medical Sciences, Lund University, Lund, Sweden; 2 Primary Health Care Region Skåne, Malmö, Sweden; 3 Lund University Diabetes Centre, Malmö, Sweden; 4 Lund University, Skåne University Hospital Lund, Lund, Sweden; 5 Medical Radiation Physics, Department of Translational Medicine, Lund University, Malmö, Sweden

**Keywords:** Fat distribution, Plasma triglycerides, Adipose tissue, Oral fat tolerance test, Ethnicity, Cardiovascular disease, Type 2 diabetes

## Abstract

**Purpose:**

Previous studies have shown that at a similar body mass index, Middle Eastern immigrants are more insulin resistant and at higher risk for type 2 diabetes (T2D) than native Europeans. Insulin resistance is strongly associated with disturbed fat metabolism and cardiovascular disease (CVD). However, fat metabolism is poorly investigated comparing Middle Eastern and European ethnicities.

**Methods:**

This observational study included 26 Iraqi and 16 Swedish-born men without T2D or clinical risk factors for CVD. An oral fat tolerance test (OFTT) was performed, where plasma triglycerides (p-TG) were measured for 6 h. mRNA expression and adipocyte size were measured in subcutaneous adipose tissue biopsies collected prior to OFTT, and magnetic resonance imaging was conducted to assess body fat distribution.

**Results:**

The median p-TG accumulation was higher and the clearance slower among Iraqis than Swedes. None of the groups reached their fasting p-TG (Iraqis 1.55 mmol/l; Swedes 0.95 mmol/l) after 6 h (Iraqis p-TG 3.10 mmol/l; Swedes p-TG 1.50 mmol/l). Adipocyte size, mRNA expression, and fat accumulation in the liver, muscle and abdomen were similar in both groups.

**Conclusion:**

Postprandial p-TG levels rather than fat distribution may reflect early signs of disturbed fat metabolism in Iraqi immigrants without CVD risk factors.

## Introduction

Type 2 diabetes (T2D) and insulin resistance are associated with an increased risk of cardiovascular disease (CVD) and mortality [1]. T2D is increasing in most areas of the world, with the Middle East (ME) having among the highest prevalence, varying between 7 and 12% [2]. Immigrants from ME constitute the largest non-European immigrant population in Sweden. Interestingly, the Iraqi immigrant population is more insulin resistant and displays relative insulin deficiency in the nondiabetic stages compared to native Swedes [3, 4]. However, the underlying mechanisms for these observations are poorly understood.

Metabolic abnormalities leading to T2D are thought to be secondary to overweight and insulin resistance [5]. Specifically, abnormal lipid metabolism in insulin resistance is characterized by elevated fasting levels of plasma triglycerides (p-TGs), a decrease in high-density lipoprotein cholesterol, and an increase in free fatty acid levels [6]. The daily impact of fasting (i.e., 10 hours without intake of food or drinks other than water) is fairly short in real life, and individuals are in the postprandial state for most of the day. Hence, postprandial, rather than fasting, p-TG may be a more informative marker for an individual’s capacity to metabolize lipids following a meal [7].

A decreased capacity to store excess energy in subcutaneous adipocytes leads to ectopic fat accumulation in the visceral fat depot, liver and peripheral muscle tissue, which in turn contributes to increased insulin resistance [8]. This notion is supported by observations that ectopic fat accumulation, rather than the amount of subcutaneous adipose tissue (SAT), predicts the future development of T2D [9]. Furthermore, an increased amount of visceral adipose tissue (VAT) serves as a marker of increased ectopic fat in other locations, including the heart [10]. Other ectopic fat depots are the intermuscular adipose tissue (IMAT), found beneath the fascia and within the muscles, and so-called intramyocellular lipids (IMCL). An increase in either IMAT or IMCL levels has been associated with insulin resistance [11, 12].

The release and storage of fatty acids, as well as the differentiation of adipocytes, is tightly regulated by insulin [13]. Adipose tissue expands by increasing both adipose cell size (hypertrophy) and cell number (hyperplasia). Previous studies have shown that enlarged adipocytes have an impaired insulin response [14, 15] and that increased adipose cell size is positively correlated with impaired systemic insulin sensitivity and glucose tolerance [16]. Furthermore, the expression and activity of adipose tissue lipoprotein lipase are affected in insulin-resistant individuals and have been proposed to contribute to a delayed clearance of postprandial and fasting p-TG levels [17].

Pronounced insulin resistance and relative insulin deficiency reflect metabolic abnormalities in the nondiabetic stages in ME immigrants. We hypothesized that healthy ME men would differ in fat tolerance and accumulation of ectopic and visceral fat compared with healthy, native Swedish men. To address these hypotheses, we compared 1) the p-TG response to a standardized fat meal, 2) adipocyte size, 3) mRNA expression of key regulators of lipid and glucose metabolism, and 4) fat distribution in nonobese, nonsmoking men born in Iraq or Sweden without a history of T2D or CVD.

## Material and methods

### Study population

The MEDIM cohort is a cross-sectional study conducted 2010–2012 including Iraqi and Swedish-born residents of Malmö, Sweden (30–75 years of age, residing in the same geographical area in Malmö, matched for age) as previously described [18]. A subset of men participating in the MEDIM study, where the health examination showed that they were never smokers, nonobese (body mass index (BMI) < 30 kg/m^2^), and without a history of hypertension, T2D, hyperlipidemia or CVD, were invited to participate in the study (either March to May 2017 or March to May 2018).

Physical activity was self-assessed using the international physical activity questionnaire (IPAC) capturing time (minutes) physically active over the last 7 days [19]. Those who were less active than 150 minutes were considered physically inactive.

Food habits were also self-assessed using National Board of Health and Welfare guidelines for methods of preventing disease [20], capturing frequency of intake of vegetables, fruit and berries, fish and seafood as well as intake of sweet food and drinks (such as bakery, chocolate, dessert, sweets, etc.). A diet index can capture the quality of food intake, and low points < 9 are considered unhealthy eating habits [20].

Participants with at least three of the following were considered to have metabolic syndrome: systolic blood pressure > 140 mmHg; diastolic blood pressure > 90 mmHg; fasting TG > 1.7 mmol/l; insulin resistance (HOMA-IR above the 4th quartile, i.e., > 2.6) or BMI > 30 kg/m^2^ [21].

All subjects completed a magnetic resonance imaging (MRI) exclusion form with standard MRI patient safety criteria. Only subjects without any contraindications for MRI (defined as noncompatible metallic implants or devices or claustrophobia) were eligible.

**Fig. 1:**
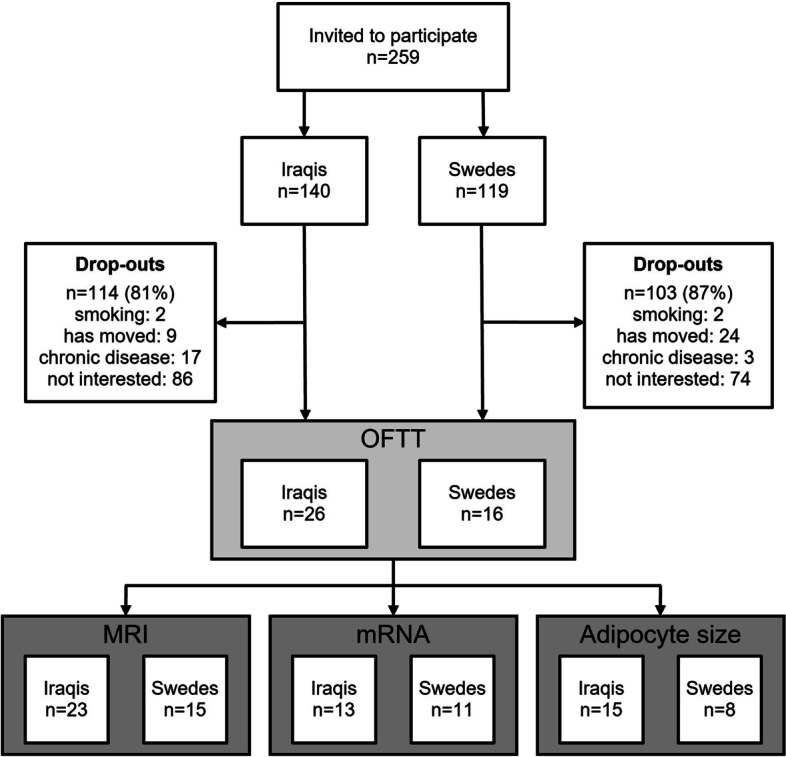
Flowchart for eligible participants in conducting the oral fat tolerance test. †Oral Fat Tolerance Test (OFTT), ‡ Magnetic Resonance Imaging (MRI)

In total, 259 nonobese males (born in Iraq *n* = 140; born in Sweden *n* = 119) without CVD risk factors were invited to participate in the study (Fig. 1). A total of 42 men, 26 Iraqi born and 16 Swedish born, participated in the study, including fasting samples and oral fat tolerance tests (OFTTs). Biopsies were conducted in 25 Iraqi and 16 Swedish-born men. In 13 Iraqi-born and 11 Swedish-born men, mRNA was assessed, and adipocyte size was measured in 15 Iraqi-born and 8 Swedish-born men.

In total, 23 men born in Iraq and 15 men born in Sweden were examined by MRI. Due to technical errors, four thigh data sets (two Swedish-born and two Iraqi-born), eight abdominal data sets (four Swedish born and four Iraqi born), and eleven spectra (five Swedish born and six Iraqi born) could not be evaluated and were therefore excluded from the MRI results.

### Laboratory assessments

Anthropometrics, body composition, and laboratory data were assessed by trained Swedish research nurses with the presence of an Arabic-, English- and Swedish-speaking interpreter. Body weight was measured using an electronic scale (Coline, 34–5062 RTC3010, China), while participants were requested to wear light clothing and remove shoes. A wall-mounted stadio-meter was used to measure height. Waist circumference was measured to the nearest cm in the standing position after gentle expiration. A tape measure was placed around the bare midriff of each participant, and the waist circumference was measured midway between the lower border of the rib cage and the superior border of the iliac crest. Hip circumference was measured at the maximum level, to the nearest cm, using a tape measure. Bioelectrical impedance analysis was used to assess fat mass and fat % (Tanita Pro, Tanita Europe BV, The Netherlands).

Fasting blood samples were collected and analyzed for blood glucose, total cholesterol, triglycerides (p-TG), high-density lipoprotein cholesterol (p-HDL) and low-density lipoprotein cholesterol (p-LDL). Homeostatic model assessment (HOMA) was used to estimate both insulin resistance (HOMA-IR) and beta cell function (HOMA-beta) [22].

Indices were calculated as follows:

HOMA - IR = fasting insulin (mU/L) × fasting glucose (mmol/L)/22.5.

HOMA - β = [20 × fasting insulin (mU/L)/[fasting glucose (mmol/L) - 3.5].

### Oral fat tolerance test (OFTT)

An OFTT was conducted after a 12-h overnight fast [7]. A standard fat meal of 4425 kJ (1057 kcal) was prepared from 100.0 g vanilla ice cream, 150.0 g cream, 70.0 g chocolate sauce, and 35.0 g natural cottage cheese, in total containing 5 energy percent (E%) protein (13.5 g), 66 E% fat (78.7 g) and 29 E% carbohydrates (74.0 g). For lacto-intolerant individuals, there was a corresponding lactose-free alternative of 4433 kJ (1059 kcal), containing 14.0 g protein, 78.5 g fat (of which 49.8 g was saturated fat) and 74.0 g carbohydrates. The participants were instructed to drink the meal within 15 minutes and were asked to keep physical activity to a minimum and not drink or eat during the following 6 h. Blood samples were drawn at baseline (fasting samples), 30 minutes, 1 h, 2 h, 3 h, 4 h and 6 h for determination of p-TG.

### Fat distribution in the liver, thigh and abdomen

To examine fat accumulation in the liver and thigh as well as the abdominal adipose tissue distribution, subjects from both groups underwent magnetic resonance imaging (MRI) and magnetic resonance spectroscopy (MRS) examinations of the liver, abdomen, and thigh using a 3 T MRI scanner (TIM Trio, Siemens Healthineers, Erlangen, Germany). To estimate the IMCL/water concentration ratio in the thigh, two point resolved spectral selection techniques (PRESS) were acquired: one with water suppression for the estimation of the IMCL signal amplitude and one without water suppression for the estimation of the water signal amplitude.

Fat/water imaging using six echoes was performed to obtain proton density fat fraction (PDFF) maps of the liver and muscle (thigh) [23]. Semiautomated approaches were used to outline the liver, SAT of the thigh, and thigh muscle regions of interest (ROIs). Using the estimated PDFF maps of the left thigh, the IMAT and SAT-thigh volumes were calculated by summing the PDFFs within the respective ROIs and multiplying by the voxel size. IMAT was defined as fat within the muscle fascia [24]. Fat-only images from the volumetric interpolated breath-hold examination (VIBE) acquisition were used to estimate the volumes of abdominal SAT and VAT. A semiautomated region-grow method was used to outline the subcutaneous depot, while the visceral depot was manually outlined to avoid the spinal cord. To further separate the VAT from other structures and organs within the abdominal cavity, a threshold was used. Only voxels above the cutoff value were considered adipose tissue and included in the VAT ROI [25, 26].

The MRS analysis was conducted using JMRUI software [27, 28]. The amplitude of IMCL was estimated from the water-suppressed spectra, while the water amplitude was assessed from the spectra without water suppression. The ratio between the concentration of IMCL and water was then calculated as suggested by Boesch et al. [29]. All the MRI and MRS sequences and corresponding settings are shown in Table 2.

### Adipocyte-size distribution and mRNA expression in subcutaneous fat

Subcutaneous adipose tissue samples were obtained through a needle biopsy following local anesthesia at the right side of the umbilicus at a distance of approximately 7–10 cm. Samples were analyzed for adipocyte-size distribution using a Beckman-Coulter counter after osmium fixation as described previously [30]. For mRNA analysis, samples were immediately snap-frozen in liquid nitrogen, lysed and homogenized in Qiazol™ lysis reagent (Qiagen). RNA was isolated using an RNeasy® Mini Kit (Qiagen) according to the manufacturer’s recommendations. RT-qPCRs were performed using the Quantifast SYBR Green RT–PCR kit (Qiagen) and Quantitect primer assays for *18S, SLC2A4, COL1A1, LPL, and CIDEA* according to the manufacturer’s instructions. Primer sequences are considered proprietary information by Qiagen. mRNA expression levels were measured using a StepOnePlus real-time thermal cycler (Applied Biosystems, Waltham, USA) and quantitated using the ΔΔC_T_ method as described by Livak and Schmittgen [31]. 18S mRNA expression levels were used for normalization.

### Statistical analyses

Analyses were performed using SPSS version 24 (IBM). Comparisons of baseline characteristics between the Swedish and Iraqi populations were assessed using the independent-sample T test or the Mann–Whitney U test for normal and nonnormally distributed continuous variables, respectively (Table 1). Data on total area under the curve (tAUC), incremental AUC (iAUC) of postprandial p-TG concentration from Iraqis and Swedes were Log_10_ transformed (i.e., residuals tested for normality) and analyzed by general linear models (GLM) with age and BMI as covariates. Two-sided *P* values < 0.05 were considered statistically significant.

**Table 1:** Baseline characteristics of study participants that underwent an oral fat tolerance test

Characteristics	Country of birth		
	Sweden (***n*** = 16)	Iraq (***n*** = 26)	Total (***n*** = 42), ***p***-values
Age (median, IQR)^a^	53.7 (64.3–39.8)	46.7 (55.5–41.9)	0.484
Body Mass Index (kg/cm2), (median, IQR)^a^	26.1 (27.1–24.1)	26.9 (28.0–25.3)	0.055
BMI < 25 (median, IQR)^a^	23.8 (24.6–22.9) *n* = 6	24.7 (24.8–24.1) *n* = 5	0.082
BMI > 25 (median, IQR)^a^	26.7 (27.7–26.2) *n* = 10	27.6 (28.2–26.6) *n* = 21	0.319
Systolic blood pressure (mmHg)^b^	127.9 (17.6)	115.2 (8.1)	0.014
Diastolic blood pressure (mmHg)^b^	70.2 (10.2)	66.6 (6.0)	0.226
Waist circumference (cm)^a^ (median, IQR)	91.0 (97.0–86.6)	95.0 (99.3–92.5)	0.153
Fasting glucose (mmol/l), (means, SD)^b^	5.86 (0.53)	5.72 (0.50)	0.372
HOMA-beta (median, IQR)^a^	55.5 (72.2–45.8)	71.7 (96.1–45.2)	0.265
HOMA-IR (median, IQR)^a^	1.67 (2.59–1.25)	2.14 (2.54–1.24)	0.543
Total cholesterol (mmol/L), (mean, SD)^b^	4.91 (0.66)	4.78 (1.09)	0.680
p-LDL (mmol/L), (mean, SD)^b^	3.35 (0.68)	3.54 (0.90)	0.466
p-HDL (mmol/L), (median, IQR)^a^	1.35 (1.50–1.23)	1.00 (1.30–0.94)	0.001
Fat % (median, IQR)^a^	23.8 (26.0–20.6)	24.7 (26.9–22.6)	0.224
Apo A1(g/L), (mean, SD)^b^	1.45 (0.20)	1.20 (0.25)	0.003
Apo B (g/L), (mean, SD)^b^	0.99 (0.18)	1.10 (0.27)	0.164
ApoA1/ApoB (mean, SD)^b^	0.70 (0.16)	1.05 (0.80)	0.088
p-Triglyceride OFTT			
Fasting p-TG (mmol/l) (median, IQR)^a^	0.95 (1.65–0.80)	1.55 (2.40–1.60)	0.104
p-TG 0.5 h (median, IQR)^a^	1.00 (1.68–0.83)	1.70 (2.50–1.03)	0.053
p-TG 1 h (median, IQR)^a^	1.25 (1.80–1.00)	2.20 (2.63–1.25)	0.027
p-TG 2 h (median, IQR)^a^	2.00 (2.38–1.45)	3.10 (4.00–1.88)	0.011
p-TG 3 h (median, IQR)^a^	2.10 (3.08–1.80)	3.55 (5.10–2.50)	0.011
p-TG 4 h (median, IQR)^a^	1.95 (3.38–1.63)	3.55 (5.95–2.40)	0.033
p-TG 6 h (median, IQR)^a^	1.50 (3.58–1.30)	3.10 (4.60–1.70)	0.049
tAUC TG mmol/L^c^min (median, IQR)^a^	601.6 (998.6–488.2)	1017.8 (1578.8–738)	0.036
iAUC TG mmol/L^c^min (median, IQR)^a^	267.0 (481.1–187.9)	495.0 (802.5–267.4)	0.011
Metabolic syndrome, % (n) ^c^	12.2 (2)	0	0.065
Physical inactivity < 150 min/week, % (n)^c^	43.8 (7)	46.2 (12)	0.879
Physical activity minutes/week ^a^	262.5 (787.5–37.5)	150.0 (540.0–26.0)	0.420
Unhealthy eating habits, % (n)^c^	56.3 (9)	57.7 (15)	0.927

^a^Data presented as medians (interquartile range, IQR), two tailed Mann-Whitney U-test

^b^Data presented as means (standard deviation, SD), independent sample t-test

^c^Data presented a percentage (numbers), chi-square test

tAUC and iAUC adjusted for age and BMI

**Table 2:** The MRI and MRS examinations and the corresponding parameter settings

MR sequence	Examined body part	Echo time(s) (ms)	Repetition time (ms)	Field of view (mm2)	Matrix size	Slice thickness (mm)	Number of slices	Voxel size (mm3)
3D GRE, breath-hold	Liver	1.51, 3.1, 4.6, 6.2, 7.8, 9.3	11	380 × 285	128 × 128	5	40	
2D GRE	Left thigh muscles, centered around middle of the thigh	1.3, 4.3, 7.3, 10.3, 13.3, 16.3, 19.3, 22.3, 25.3, 28.3, 31.3, 34.3	243	350 × 252	192 × 192	5	5	
VIBE, breath-hold	Abdomen (L3-L4 spinal disc)	1.2, 2.5	4	400 × 325	320 × 320	5	32	
PRESS	Semitendinosus muscle	30	3000					12x12x30

Wilcoxon rank sum tests were conducted to compare mRNA expression, MRI-estimated PDFFs, adipose tissue volumes, and IMCL/water concentrations of the Iraqi-born men to those of the Swedish-born men. The adipose cell size distribution is illustrated by plotting the average frequency within each bin measured from all subjects in each group (Iraqi versus Swedish). Maximum adipocyte size was collected at the peak value in the large adipose cell population for each individual.

## Results

### Baseline characteristics

There were no differences in lifestyle habits between the groups (Table 1). However, Swedish-born men presented with higher systolic blood pressure than Iraqi-born men. Furthermore, two Swedish but no Iraqi men fulfilled the criteria for metabolic syndrome. No differences in baseline characteristics were observed between the two groups, except for lower HDL and ApoA1 levels in Iraqi compared to native Swedish participants (Iraqis 1.00 mmol/l HDL, Swedes 1.35 mmol/l HDL, *P* = 0.001; Iraqis 1.20 g/l ApoA1, Swedes 1.45 g/l ApoA1, *P* = 0.003) (Table 1). No adjustments were made since the groups were well matched.

### Oral fat tolerance test (OFTT)

**Fig. 2:**
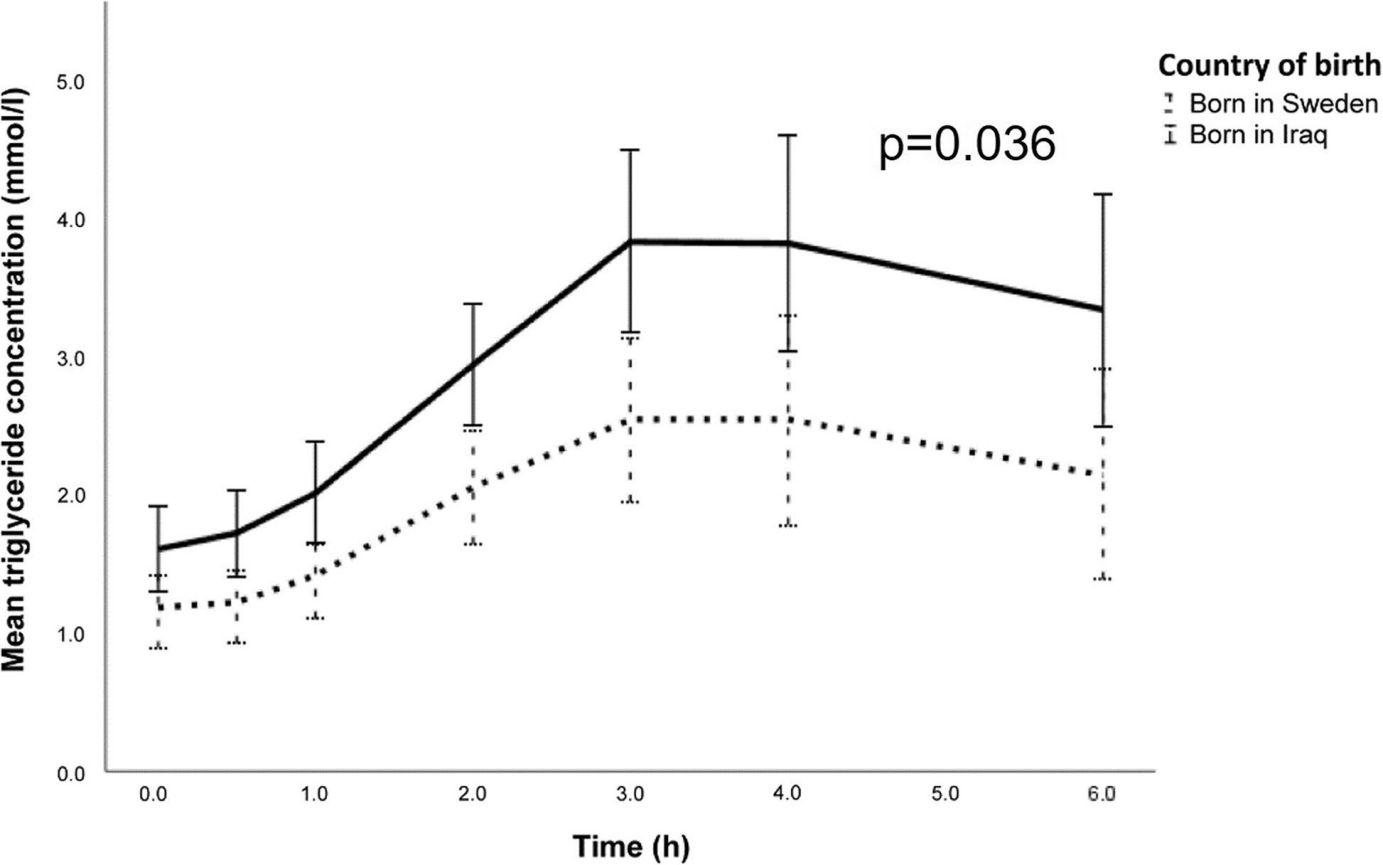
Plasma triglyceride concentrations during an oral fat tolerance test (OFTT). **a** Comparison of Iraqi immigrants and native Swedes assessed as estimated marginal means (95% confidence interval). **b** Same as in a, restricted to participants with BMI 25–30 kg/m^2^. a-b) Blue lines represent Iraqis, purple lines represent Swedes

The median fasting p-TG values were within the normal range at baseline (< 1.7 mmol/l) and peaked at 3 h post-ingestion in both groups (Fig. 2). However, during the 6-h load, the p-TG accumulation was generally higher in the Iraqi born group, with higher p-TG concentrations at 2 h, 3 h, 4 h and 6 h, higher tAUC and iAUC than in the Swedish born group (Table 1, Fig. 2). Six hours after ingestion, none of the groups had reached their fasting p-TG value (Iraqis 3.10 mmol/l versus Swedes 1.50 mmol/l, *P* = 0.049 (Table 1).

### Fat distribution in the liver, thigh and abdomen

**Fig. 3:**
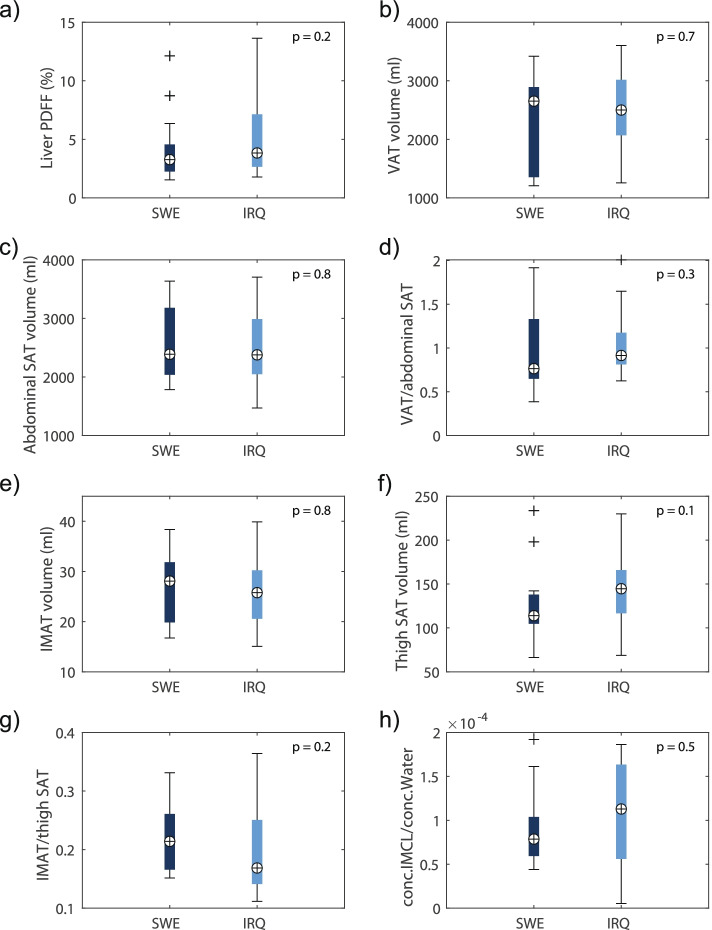
Comparison of fat accumulation in various fat depots between Iraqi immigrants and native Swedes, estimated with magnetic resonance imaging (MRI). Boxplots of the magnetic resonance imaging (MRI)-estimated **a** liver proton density fat fraction (PDFF), **b** visceral adipose tissue (VAT) volume, **c** abdominal subcutaneous adipose tissue (SAT) volume and **d** VAT/abdominal SAT ratio. Shown are the boxplots of the estimated **e** intermuscular adipose tissue (IMAT) volume, **f** thigh SAT volume, **g** IMAT/thigh SAT ratio, and h) ratio between the concentration of intramyocellular lipids (IMCL) and water of the semitendinosus muscle of the left thighs

**Fig. 4:**
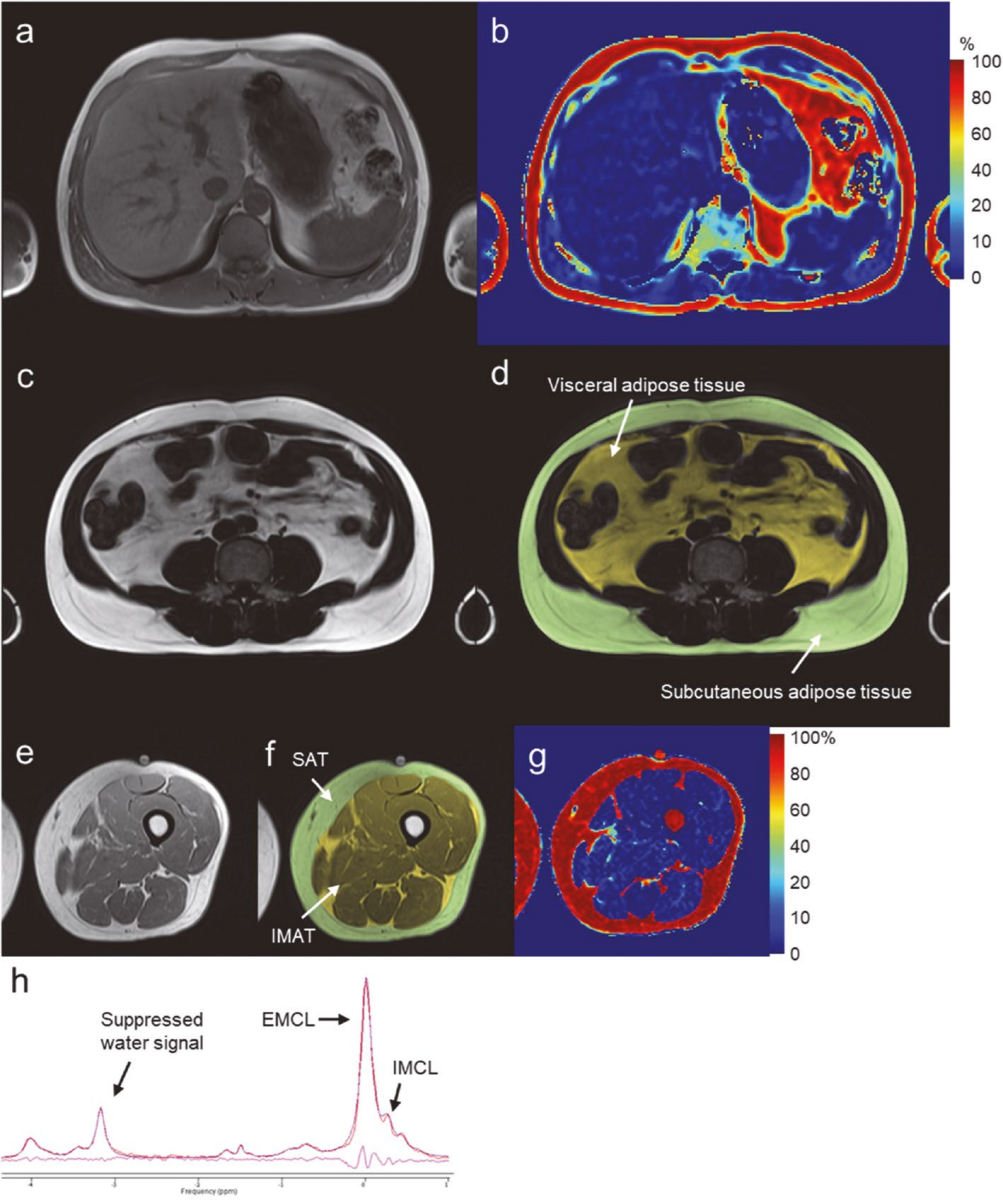
Example of MRI imaging. Examples of **a** acquired liver VIBE (in-phase image), **b** calculated liver PDFF, **c** acquired abdominal VIBE (fat image), **d** visceral (yellow) and subcutaneous (green) adipose tissue ROIs, **e** acquired thigh VIBE (in-phase image), **f** subcutaneous adipose tissue (green) and IMAT (yellow) ROIs, **g** calculated thigh PDFF images, and **h** a water-suppressed PRESS spectrum of the semitendinosus muscle

MRI measurements revealed no difference in liver PDFF (3.84% vs. 3.27%, *P* = 0.2), VAT volume (2500 mL vs. 2652 mL, *p* = 0.7), or abdominal SAT volume (2378 mL vs. 2387 mL, *P* = 0.8) between Iraqis and Swedes (Fig. 3a-c). Examples of MRI and MRS data and corresponding ROIs are presented in Fig. 4. Furthermore, both IMAT volumes (28 mL vs. 26 mL, *P* = 0.8) and SAT volume in thighs (114 ml versus 145 ml, *P* = 0.1) were similar between Iraqis and Swedes (Fig. 3e-f). Likewise, no difference was found in the concentration of IMCL and water ratio estimated with MRS (0.8*10^− 4^ vs. 1.3*10^− 4^, *P* = 0.5) (Fig. 3h) or the ratio between VAT and abdominal SAT (0.91 versus 0.76, *P* = 0.3) and IMAT and thigh SAT (0.17 versus 0.21, *P* = 0.2) (Fig. 3d and g).

### Adipocyte-size distribution and mRNA expression in subcutaneous fat

**Fig. 5:**
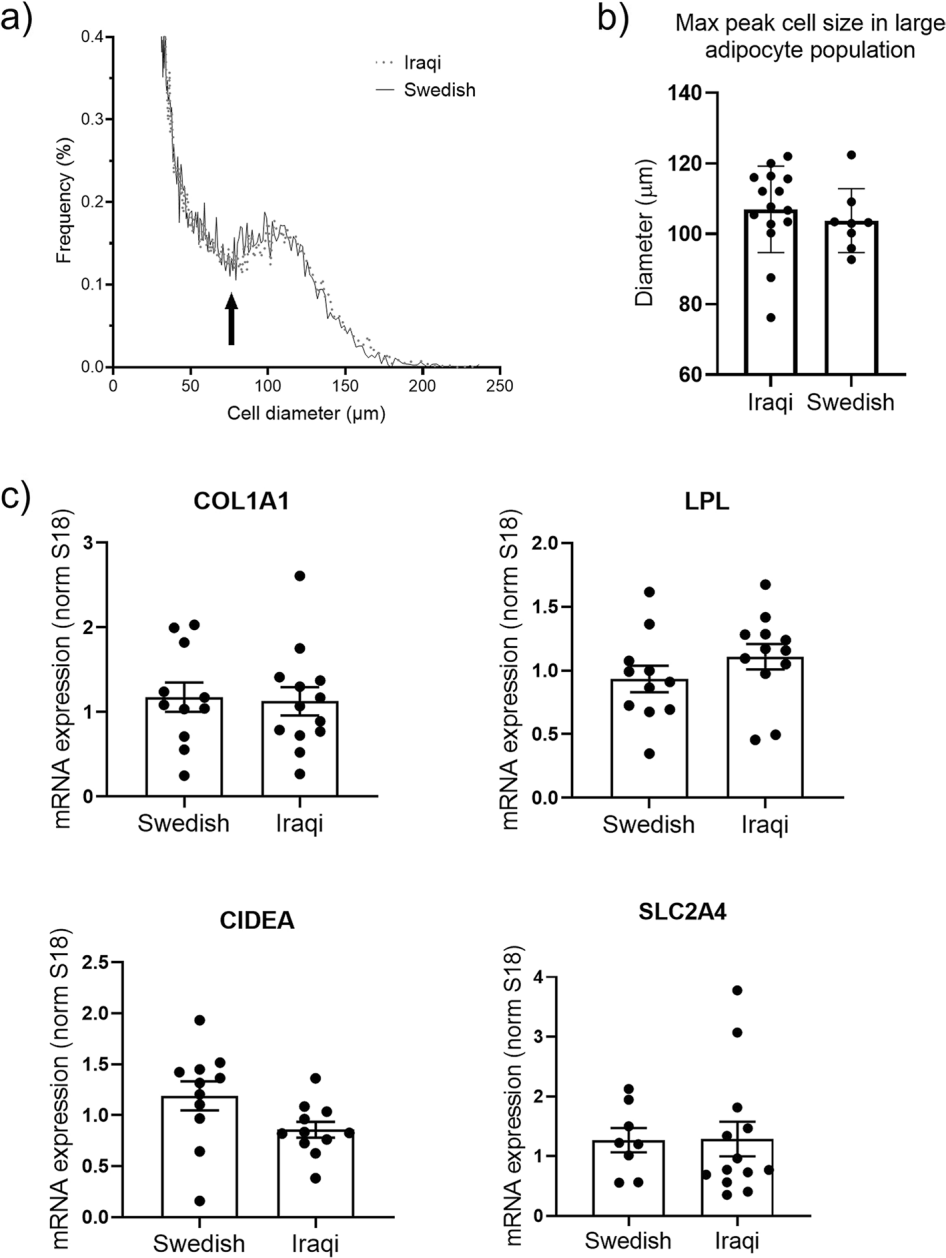
Subcutaneous adipose tissue analyses. The adipocyte-size distribution of fixed, intact subcutaneous adipose tissue was measured using a coulter counter, ~ 6000 counts/sample, and each sample was run in duplicate. **a** The graph displays the average distribution curves from *n* = 15 Iraqi and *n* = 8 Swedish subjects. **b** The fraction of small cells is defined to the left of the nadir (arrow), and the fraction of large cells is defined to the right of the nadir. **c** mRNA expression of *COL1A1*, *LPL*, *SLC2A4*, and *CIDEA*, measured by qPCR, *n* = 11–13 Iraqi and *n* = 8–11 Swedish subjects. Data are normalized to *18S* expression

Cell-size analysis revealed a bimodal adipocyte-size distribution in both the Swedish and Iraqi born groups (Fig. 5a), with a fraction of small cells, defined by the nadir [32], which is the lowest point between the two cell populations and a fraction of large cells (right of the nadir). The distribution curves were similar between Iraqi and Swedes (Fig. 5a), where the large cell fraction ranged between 75 and 200 μm, with a mean peak cell size of ~ 105 and 103 μm (Iraqis and Swedes, respectively) (Fig. 5b). Furthermore, we observed no differences in the mRNA expression of key regulators of lipid and glucose metabolism (*SLC2A4, COL1A1, LPL, CIDEA*), but there was a trend toward higher mRNA expression of *LPL* in Iraqi individuals (Fig. 5c).

## Discussion

In the current study, we show that the Iraqi-born participants had higher postprandial TG levels than Swedish-born participants, even though participants in both groups were nonobese (BMI < 30 kg/m^2^) and nondiabetic. Information about postprandial TG levels between ethnic groups is scarce [33–35], and to the best of our knowledge, our study is the first to compare postprandial TG between men of Middle Eastern and European ancestry. Bower et al. found that lean African American women had lower fasting p-TG levels as well as low postprandial p-TG levels compared to lean Caucasians; however, no differences were seen between obese African American and obese Caucasians in p-TG levels [33]. When comparing young black and white males, the overall postprandial p-TG responses were similar between the groups, although white men showed a higher incremental response during two to 4 h following the fat load [35]. Furthermore, the lipid response to a high-fat meal did not differ between South Asians, Latin Americans and Northern Europeans in a small pilot study [34]. On the other hand, there are several studies focusing on the effects of OFTT without analyzing the influence of ethnicity. In these studies, postprandial hypertriglyceridemia was prolonged in obese adults with high insulin resistance [36], as well as in young obese individuals [37] and in patients with T2D [38]. These findings suggest that elevated postprandial p-TG is associated with metabolic disturbances and that there are indices of differences in p-TG response between ethnicities.

The concentration of intestinal and hepatic TG-rich lipoproteins increases in the postprandial phase [39, 40]. Future studies are needed to investigate whether the increased TG levels in Iraqi men are explained by slower clearance or rather by increased lipoprotein production.

Abdominal obesity and overweight have been associated with postprandial p-TG lipaemia [36, 37]. Despite the observed difference in levels of TG, we found no differences in body fat distribution, fat-cell size or expression of genes encoding proteins involved in glucose and lipid handling in subcutaneous fat between the Swedish and Iraqi groups. Thus, we could not detect a difference in lipid storage capacity and adipose tissue function between Iraqis and Swedes. However, in line with increased p-TG levels following OFTT, Iraqis had a trend toward increased *LPL* mRNA levels in subcutaneous adipose tissue. This could be part of a compensatory mechanism trying to overcome an impaired capacity of transporting lipids from the circulation into the adipocytes. Nevertheless, the difference in p-TG levels between Iraqis and Swedes remained when adjusting for age and BMI. Furthermore, it is well established that a high VAT mass can predict cardiometabolic risk [11], and the VAT/SAT ratio has been suggested as an even more reliable predictor than the amount of VAT alone [41]. Our MRI analysis revealed no differences in either adipose tissue content in any of the measured depots. Likewise, no differences in fat mass or body fat % were found by impedance measurements. Thus, our findings suggest that Iraqis may have a metabolic (fat) phenotype that is distinct from Caucasians, and further studies in larger cohorts are warranted to corroborate this notion.

The p-TG level fluctuates substantially with food intake, depending on differences in lipoprotein production, clearance, and the amount and content of the regular food intake. In recent data from the MEDIM randomized controlled trial assessing a self-reported dietary diary, Iraqi immigrants generally obtained > 40 E% from fat compared to 34 E% in Swedes [42]. It is not inconceivable that the high-fat diet in the Iraqi population is a promotor for insulin secretion and subsequent earlier development of insulin resistance and T2D [3]. Indeed, we have previously reported that the nondiabetic Iraqi born Middle Eastern immigrant population is more insulin resistant than the background native Swedish population [3]. During the development of T2D, Iraqis display, to a higher extent, severe insulin deficiency compared to native Swedes [43]. This suggests that high insulin resistance in the nondiabetic stage contributes to relative insulin-deficient T2D and different diabetes phenotypes [43]. Taken together, progression toward insulin resistance and T2D may therefore not be preceded by an altered fat distribution or adipocyte hypertrophy [44, 45] but rather elevated postprandial p-TG levels. Moreover, recent studies have shown that ApoA1 can stimulate glucose uptake in peripheral tissues in a noninsulin-dependent manner [46]. Hence, decreased HDL/ApoA1 levels, as observed in the current study, may increase the risk of developing insulin resistance among the Iraqis.

Previous studies suggest that increased nonfasting p-TG levels could be a more sensitive risk factor for cardiovascular disease than fasting p-TG levels due to sustained and prolonged hypertriglyceridemia [47]. Interestingly, the OFTT unmasked changes in lipid metabolism between Iraqis and Swedes only noticeable in the postprandial state. Therefore, we propose that the OFTT may be a way to identify metabolically healthy Iraqis at risk of developing metabolic disturbances.

## Limitations

The main strengths of our study are the well-defined populations from the same sociodemographic areas and that the groups are well balanced in anthropometrics. In addition, our study is based on an OFTT, which has been shown to better reflect postprandial p-TG than other methods [7]. Limitations involve the relatively small study population with fewer participants born in Sweden. Furthermore, our study was based on young and middle-aged men, and the results may therefore not be generalizable to other populations.

## Conclusion

In conclusion, we found that despite having normal fasting p-TG levels and similar body fat distribution, healthy Iraqi male immigrants conducting an OFTT displayed a high p-TG peak and a delayed postprandial p-TG clearance compared with Swedes. Our findings pinpoint differences in fat metabolism across ethnicities, which needs to be further investigated in a larger study.

## Data Availability

The raw data supporting the conclusions of this article will be made available upon request by the authors, with undue reservation.

## Abbreviations

BMI: Body mass index
HOMA: Homeostasis model assessment
iAUC: Incremental area under the curve
IMCL: Intramyocellular lipids
IMAT: Intermuscular adipose tissue
ME: Middle eastern
MRI: Magnetic resonance imaging
MRS: Magnetic resonance spectroscopy
OFTT: Oral fat tolerance test
PDFF: Proton density fat fraction
p-TG: Plasma triglycerides
p-HDL: Plasma high-density protein
p-LDL: Plasma low-density protein
ROI: Region of interest
SAT: Subcutaneous adipose tissue
VAT: Visceral adipose tissue
tAUC: Total area under the curve
T2D: Type 2 diabetes
